# CircEAF2 counteracts Epstein-Barr virus-positive diffuse large B-cell lymphoma progression via miR-BART19-3p/APC/β-catenin axis

**DOI:** 10.1186/s12943-021-01458-9

**Published:** 2021-12-01

**Authors:** Chen-xing Zhao, Zi-xun Yan, Jing-jing Wen, Di Fu, Peng-peng Xu, Li Wang, Shu Cheng, Jian-da Hu, Wei-li Zhao

**Affiliations:** 1 Fujian Institute of Hematology, Fujian Provincial Key Laboratory of Hematology, Fujian Medical University Union Hospital, 29 Xinquan Road, Fuzhou, 350001 Fujian China; 2 Shanghai Institute of Hematology, State Key Laboratory of Medical Genomics, National Research Center for Translational Medicine at Shanghai, Ruijin Hospital Affiliated to Shanghai Jiao Tong University School of Medicine, 197 Rui Jin Er Road, Shanghai, 200025 China; 3 Pôle de Recherches Sino-Français en Science du Vivant et Génomique, Laboratory of Molecular Pathology, Shanghai, China

**Keywords:** Epstein-Barr virus, Diffuse large B-cell lymphoma, circEAF2, miR-BART19-3p, Wnt signaling pathway, β-catenin

## Abstract

**Background:**

Epstein-Barr virus (EBV) represents an important pathogenic factor of lymphoma and is significantly associated with poor clinical outcome of diffuse large B-cell lymphoma (DLBCL). Circular RNAs (circRNAs) play an essential role in lymphoma progression. However, the underlying mechanism of circRNA on DLBCL progression related to EBV remains largely unknown.

**Methods:**

CircRNA was screened by high-throughput sequencing in tumor samples of 12 patients with DLBCL according to EBV infection status. Expression of circEAF2, as well as the relationship with clinical characteristics and prognosis, were further analyzed in tumor samples of 100 DLBCL patients using quantitative real-time PCR. Gain- and loss-of-function experiments were conducted to investigate the biological functions of circEAF2 both in vitro and in vivo. The underlying mechanism of circRNA on DLBCL progression were further determined by RNA sequencing, RNA pull down assay, dual-luciferase reporter assay, rescue experiments and western blotting.

**Results:**

We identified a novel circRNA circEAF2, which was downregulated in EBV + DLBCL and negatively correlated with EBV infection and DLBCL progression. In EBV-positive B lymphoma cells, circEAF2 overexpression induced lymphoma cell apoptosis and sensitized lymphoma cells to epirubicin. As mechanism of action, circEAF2 specifically targeted EBV-encoded miR-BART19-3p, upregulated APC, and suppressed downstream β-catenin expression, resulting in inactivation of Wnt signaling pathway and inhibition of EBV + DLBCL cell proliferation. In EBV-positive B-lymphoma murine models, xenografted tumors with circEAF2 overexpression presented decreased Ki-67 positivity, increased cell apoptosis and retarded tumor growth.

**Conclusions:**

CircEAF2 counteracted EBV + DLBCL progression via miR-BART19-3p/APC/β-catenin axis, referring circEAF2 as a potential prognostic biomarker. Therapeutic targeting EBV-encoded miRNA may be a promising strategy in treating EBV-associated lymphoid malignancies.

**Supplementary Information:**

The online version contains supplementary material available at 10.1186/s12943-021-01458-9.

## Introduction

Epstein-Barr virus (EBV) is an important human oncogenic virus and significantly related to pathogenesis and progression of diffuse large B-cell lymphoma (DLBCL) [1]. Of note, EBV is the first virus reported to encode microRNAs (miRNAs) [2], which are derived from two viral genome segments, BamHI fragment H rightward open reading frame 1 (BHRF1) and Bam HI-A region rightward transcript (BART) [3]. Recent study has shown that these EBV-encoded miRNAs contribute to B-cell transformation and lymphomagenesis [4]. Functionally, miR-BHRF1-2 represses the transcriptional activity of PRDM1, a master regulator of B-cell terminal differentiation, to promote EBV lymphomagenesis [5]. MiR-BARTs also play a crucial role in B-cell lymphoma progression. MiR-BART3, miR-BART9, and miR-BART17-5p act as the post-transcriptional regulators of BCL6 expression in DLBCL [6]. Given the abovementioned roles of EBV-encoded miRNAs, targeting EBV as a therapeutic approach appears to be promising in DLBCL.

Circular RNAs (circRNAs) are formed as closed loops by precursor RNA splicing. Owing to their circular structure to avoid degradation by RNases, circRNAs are highly stable in cells [7]. With the development of high-throughput sequencing and in-depth mechanistic researches, circRNAs have been increasingly recognized as modulators of pathological processes in lymphomas [8]. For instance, circLAMP1 enhances T-cell lymphoblastic lymphoma cell proliferation and inhibits cell apoptosis by regulating miR-615-5p/DDR2 axis [9], while circAPC sponges miR-888, initiates APC translation, and counteracts DLBCL progression [10]. CircRNAs may thus function as oncogenes or tumor suppressors, respectively, by directly sponging miRNAs or modulating their target genes. However, it is still unknown how circRNA interacts with EBV-encoded miRNAs for the pathogenesis and progression of DLBCL.

In the present study, we performed circRNA high-throughput sequencing in DLBCL according to EBV infection status and identified that a novel circRNA circEAF2 was negatively correlated with EBV infection and DLBCL progression. Meanwhile, the biological functions of circEAF2 on tumor progression were evaluated both in vitro and in vivo. Mechanistic analysis showed that circEAF2 specifically targeted EBV-encoded miR-BART19-3p, upregulated APC, suppressed downstream β-catenin expression, and counteracted EBV + DLBCL progression.

## Patients and methods

### Patients

A total of 100 patients with newly diagnosed DLBCL were enrolled in this study. The histological diagnosis was established according to World Health Organization (WHO) classification. All patients were treated by R-CHOP (rituximab combined with cyclophosphamide, doxorubicin, vincristine and prednisone)-based immunochemotherapy. The study was approved by the Shanghai Rui Jin Hospital Review Board with informed consent obtained from all subjects in accordance with the Declaration of Helsinki.

### Cell lines

Human B-lymphoma cell lines (Farage, SU-DHL-4, OCI-LY-10, OCI-LY-7, Toledo), and EBV-producing marmoset B-cell line B95-8 were purchased from ATCC (American Type Culture Collection. Manassas, VA, USA). Cells were cultured in RPMI-1640 (Gibco, Shanghai, China) and humidified atmosphere of 95% air and 5% CO_2_ at 37 °C. HEK-293 T was cultured in DMEM (Gibco, Shanghai, China). EBV-infected human B-lymphoma cell models were established using the method previously described [11]. Briefly, EBV virion was prepared from EBV-producing B95-8 cells. SU-DHL-4 and OCI-LY-10 cells were exposed to the supernatant of B95-8 cells at 37 °C for acute EBV infection (within 4 days) or long-term EBV infection (for 3 weeks). SU-DHL-4 and OCI-LY-10 cells cultured in EBV-free growth medium were used as negative controls.

### EBV DNA quantification and EBER in-situ hybridization

DNA was extracted from frozen tumor samples and cell lines using the QIAamp DNA Mini Kit (Qiagen, Valencia, CA, USA). Quantification of EBV-specific sequences was performed by real-time quantitative PCR with 7500HT Fast Real-time PCR system (Applied Biosystems, Foster City, CA, USA) using EBV PCR Fluorescence Quantitative Diagnostic Kit (DaAn Gene Co, Sun Yat-sen University, Guangzhou, China). The copy number of EBV DNA in each sample was calculated from a standard curve with a cut-off value of 700 copies/ml in tumor samples [12]. The primer sequences were obtained from DaAn Gene Co. and covered by patent. EBER (EBV-encoded RNA) in-situ hybridization was performed on 3 μm paraffin sections with 20% cut-off value of EBER-positive cells [13].

### CircRNA-Seq high-throughput sequencing

CircRNA-Seq high-throughput sequencing and subsequent bioinformatics analysis were performed by Cloud-Seq Biotech (Shanghai, China). Total RNA was used to prepare the CircRNA sequencing library by circRNA Enrichment Kit (Cloud-seq Biotech, Shanghai, China). RNA libraries were constructed by using pretreated RNAs with TruSeq Stranded Total RNA Library Prep Kit (Illumina, San Diego, CA, USA) and quality controlled by the BioAnalyzer 2100 system (Agilent Technologies, Santa Clara, CA, USA). Paired-end reads were harvested from Illumina HiSeq 4000 sequencer and quality controlled by Q30. The reads were aligned to the reference genome/transcriptome with STAR software and circRNAs were detected with DCC software and annotated by circBase database [14].

### RNA extraction, reverse transcription, and quantitative real-time PCR

Total RNA was isolated from tumor samples and cell lines using TRIzol reagent (Invitrogen, Life Technologies, Carlsbad, CA, USA) and genomic DNA (gDNA) were isolated using FastPure DNA Isolation (Vazyme, Nanjing, China). For circRNA and mRNA, reverse transcriptions were performed using the PrimeScript RT Master Mix (Takara, Shiga, Japan) with random primers. For miRNA, reverse transcriptions were performed using the PrimeScript RT Reagent Kit (Takara) with specific stem-loop primers and cDNA amplification performed using TB Green Premix Ex Taq II (Takara). GAPDH and U6 were used as internal control and each sample was repeated in triplicate. The relative fold-change in expression with respect to a control sample was calculated by the 2-ΔΔCt method. Divergent primers were used to detect backsplice junction of circRNA and convergent primers were used to detect linear mRNA. The primers for miR-BART19-3p and U6 small nuclear RNA were obtained from RiboBio Company (Guangzhou, China). The primers were listed in Table S1.

### CircRNA plasmid construction and transfection

To construct circEAF2-overexpressing plasmids, circEAF2 cDNA, as well as a specific flanking sequence of upstream and downstream (Listed in Fig. S1A), were synthesized and cloned into the pCDH-CMV-EF1а-GFP-T2A-puro vector (Lingke Biotech, Shanghai, China). Plasmids were transfected into HEK-293 T cells to package lentivirus using Lipofectamine 3000 reagent (Invitrogen). Farage and EBV-infected SU-DHL-4 cells were infected with the packaged lentivirus and then selected with 2 μg/ml puromycin (Sigma, St. Louis, MO, USA) for 14 days until circEAF2 was stably overexpressed. Circularization of the circEAF2 lentiviral construct was further validated in Fig. S1. MiRNA mimics, miRNA mutants, as well as siRNA targeting circEAF2 and control siRNA, were purchased from GenePharma (Shanghai, China) and transfected with Lipofectamine 3000 reagent (Invitrogen). The oligos were shown in Table S2.

### RNase R and actinomycin D treatment

RNase R treatment was used to identify and confirm the circRNA. Total RNA (2 μg) was incubated for 30 min at 37 °C with 3 U/μg of RNase R (Epicenter, Madison, WI, USA). Transcription was prevented by the addition of 2 μg/ml actinomycin D (Glpbio, Montclair, CA, USA) for indicated time. After treatment with actinomycin D and RNase R, RNA expression levels of circEAF2 and EAF2 mRNA were detected by qRT-PCR.

### Nucleic acid electrophoresis

The qRT-PCR products of cDNA and gDNA were investigated using 2% agarose gel electrophoresis with TAE running buffer. DNA was separated by electrophoresis at 100 V for 30 min. The DNA marker was DL2000 DNA Marker (2000-100 bp) (Takara). The bands were examined by UV irradiation.

### Luciferase report assay

The sequences of miR-binding site of circEAF2 or 3′-UTR of APC, as well as their corresponding mutants, were designed, synthesized, and inserted into luciferase reporter vector pmirGLO (Lingke Biotech). HEK-293 T cells were seeded into 24-well plates and transfected with luciferase reporter vector and miRNA mimics using the Lipofectamine 3000 reagent. After 48 h incubation, the Firefly and Renilla luciferase activities were quantified with a dual-luciferase reporter assay (Promega, Madison, WI, USA). The groups transfected wild-type circEAF2 luciferase reporter without miRNA mimics and scramble mimics were used as control group (Ctrl) and negative control group (NC), respectively.

### RNA pull-down assay

The pull-down assay was performed using Pierce Magnetic RNA-Protein Pull-Down Kit as previously described [15]. In brief, Farage cells that stably expressed circ-EAF2 were harvested, lysed, and sonicated. To pull down miRNA by circRNA, biotinylated circ-EAF2 probe was synthesized by Cloud-Seq Biotech, and oligo probe was referred as a control. After 2 h incubation with magnetic beads, cell lysates were incubated with the probes overnight. After incubation, the bound RNAs were washed and purified for further analysis. To pull down circRNA by miRNA, biotinylated miRNA mimics or their mutants were synthesized by Cloud-Seq Biotech and incubated with magnetic beads. Cell lysates were incubated with the probes overnight, and bound RNAs were detected for the abundance of circEAF2 by qRT-PCR. The sequences of probes were shown in Table S3.

### Cell proliferation and in vitro chemosensitivity assay

B lymphoma cells were seeded into 96-well plates at an initial density of 1 × 10^4^ cells per well and incubated with the indicated concentrations of epirubicin at 37 °C. After incubation, cell counting Kit-8 (CCK-8; Dojindo, Japan) was added and the absorbance of each well was measured at 450 nm by spectrophotometry. Cell proliferation curves were plotted using fold change of the absorbance at each time point and the 50% inhibitory concentration (IC50) values of epirubicin were calculated using Graphpad.

### Cell apoptosis assay

Apoptosis assay was performed using Annexin V-PE Apoptosis Detection Kit (Invitrogen) and TUNEL Cell Apoptosis Detection Kit (Beyotime, Shanghai, China) according to the manufacturer’s protocol. In brief, either transfected Farage or EBV-infected SU-DHL-4 cells (4 × 10^5^ per well) were treated with binding buffer with 5 μl Annexin V-PE and analyzed for apoptosis rates by flow cytometry (BD Biosciences, San Jose, CA, USA). Apoptotic cells were stained with 50 μL of TUNEL for 30 min at room temperature and captured under a fluorescence microscope (Leica, Wetzlar, Germany).

### RNA sequencing

RNA sequencing was downloaded from the National Omics Data Encyclopedia (NODE, http://www.biosino.org/node) under accession number OEP0001143 [16]. The raw RNA sequencing reads were aligned to human reference genome hg19 using Hisat2 (v2.0.5) and STAR (v2.5.2b). Transcript counts table files were generated by the HTSeq. Package ‘limma’ (v3.38.3) was used to normalize the raw reads and obtain differentially expressed genes (DEGs). Normalized reads were analyzed by the package ‘clusterProfiler’ (v3.10.1) and gene set enrichment analysis (GSEA) was performed. Metascape, a gene-list analysis tool, was used to verify the biological process enrichment of DEGs associated with EBV infection and low circEAF2 expression [17]. CatRAPID, an algorithm to estimate the binding propensity of protein-RNA pairs, was used to predict the binding site between targeted proteins and parental EAF2 transcript [18].

### Western blotting

Cytoplasmic and nuclear proteins were extracted from cells using BeyoECL Plus Nuclear and Cytoplasmic Protein Extraction Kit (Beyotime) supplemented with protease inhibitors and phosphatase inhibitors. Western blotting was performed as previously described [11]. Primary antibodies specific to GAPDH (1:1000 dilution, Cell Signaling Technology, Danvers, Massachusetts, USA), H3 (1:1000 dilution, Cell Signaling Technology), and APC (1:1000 dilution, Abcam, Cambridge, UK) were used. The blots were then incubated with goat anti-rabbit or anti-mouse secondary antibody (1:3000 dilution, Abcam) and visualized by Immobilon Western Chemiluminescent HRP Substrate (Millipore, St. Louis, MO, USA).

### In vivo tumorigenesis assay

Female NOD-SCID mice (4 weeks old) were randomly divided into two groups, with ten mice in each group. Farage cells stably transfected with circEAF2 overexpression plasmids or control vectors (3 × 10^6^) were injected subcutaneously into the right flank of mice randomly. Treatments were started after the tumor volumes reached about 0.5 × 0.5 cm. The untreated group received an equal volume of PBS, whereas the epirubicin group received epirubicin (1.50 mg/kg) twice weekly for 2 weeks [19, 20]. Tumor volumes were calculated as 0.5 × a (length) × b (width)^2^.

### Immunohistochemical staining

After 2 weeks, the tumors were dissected and fixed in 10% formalin. Tumor tissues were cut into 4 μm sections. The slides were treated for antigen retrieval and incubated with the Ki-67 antibody (1:100 dilution, Dako, Glostrup, Denmark) overnight at 4 °C, following by HRP-conjugated secondary antibody (1:3000 dilution, Dako) for 2 h at room temperature.

### Statistical analysis

Difference of circEAF2 expression among groups was calculated using Mann-Whitney U test. Survival curves were plotted using Kaplan-Meier method and compared by log-rank test. The Cox proportional hazards regression model was constructed for univariate and multivariate analysis. In vitro experimental results were expressed as mean ± S.D. of data obtained from three separate experiments and determined by t-test to compare variance. Statistical significance was defined as *P* < 0.05.

## Results

### Identification and characterization of circEAF2 in EBV + DLBCL

**Fig. 1:**
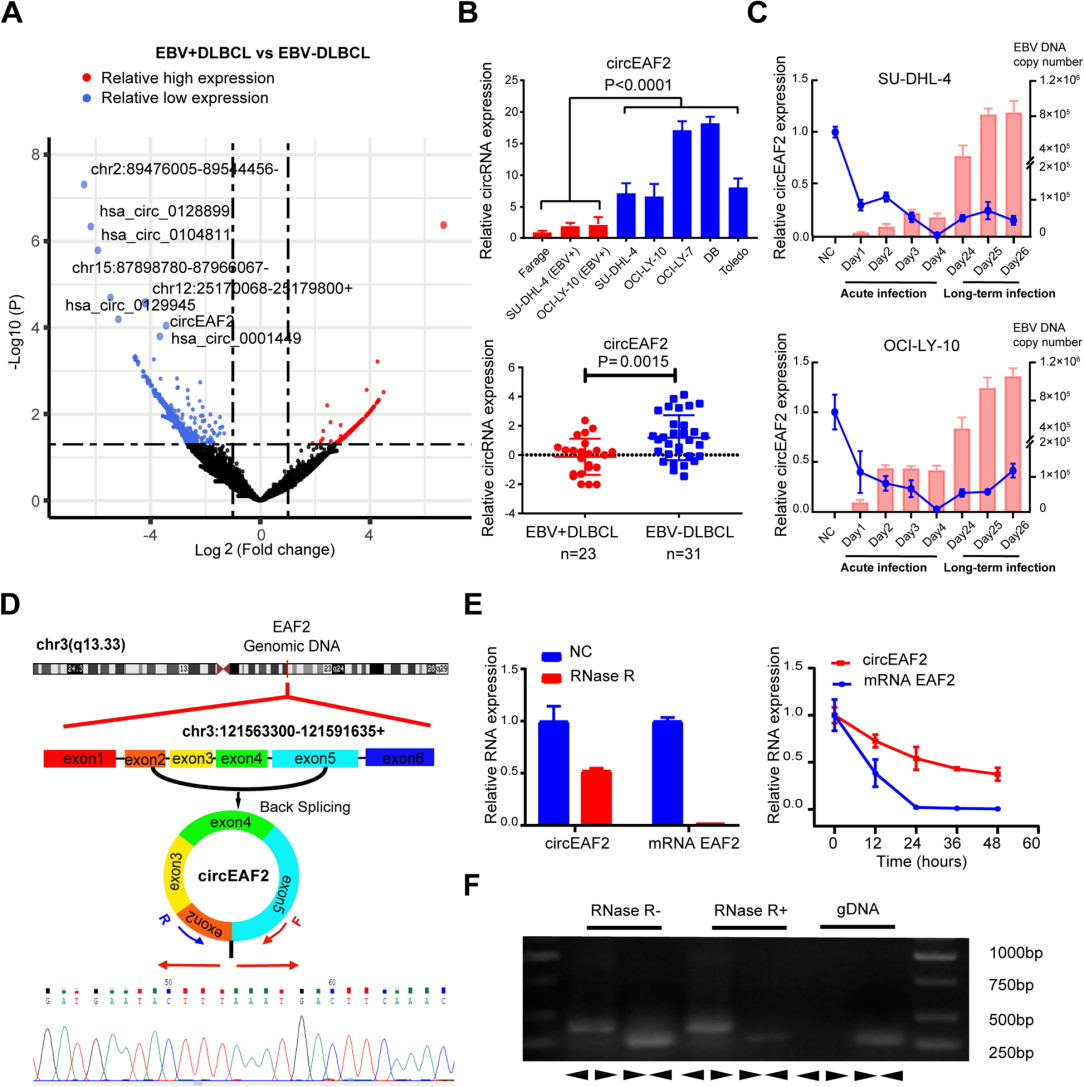
Identification and characterization of circEAF2 in Epstein-Barr virus-positive diffuse large B cell lymphoma. **A** Volcano plot showed the differential expressed circRNAs of EBV + DLBCL (*N* = 8), as compared to EBV-DLBCL (*N* = 4), analyzed by circRNA high-throughput sequencing. **B** Relative expression of circEAF2 from 7 B-lymphoma cell lines and 54 DLBCL tumor samples. Results on tumor samples was further transformed by LogE. **C** Relative expression of circEAF2 (line graph) and EBV DNA copy number (bar graph) in SU-DHL-4 and OCI-LY-10 cells during acute EBV infection or long-term EBV infection, as compared to those without EBV infection (negative control, NC). **D** Schematic diagram showed the genomic locus of circEAF2 in EAF2 gene. Sequencing results showed the back-splice junction sequences of circEAF2. Arrows represented divergent primers that bind to the back-splicing region of circEAF2. **E** Left panel: The expression of circEAF2 and mRNA EAF2 after RNase R treatment. Right panel: The expression changes of circEAF2 and mRNA EAF2 at the corresponding time points after treatment with amphotericin D. Results are presented as the RNA expression level relative to those without RNase R or amphotericin D treatment (negative control, NC). **F** qRT-PCR assay with divergent or convergent primers indicated the existence of circEAF2 or mRNA EAF2, respectively, using cDNA and gDNA as templates. EBV, Epstein-Barr virus; DLBCL, diffuse large B-cell lymphoma; circRNA, Circular RNA; EBV+, EBV positivity; EBV-, EBV negativity; R, Reverse primer; F, Forward primer; gDNA, Genomic DNA; cDNA, Complement deoxyrubonucleic acid. Con, control group. Data were shown as the mean ± S.D. of three experiments

We first performed circRNA high-throughput sequencing in frozen tissue samples of 12 DLBCL patients, including 8 EBV + DLBCL and 4 EBV-DLBCL, as previously defined [12]. In total, 107 circRNAs were found significantly downregulated in EBV + DLBCL (*P* < 0.05). To identify potentially functional circRNAs, more stringent filtering criteria (FDR < 0.1, *P* < 0.01, Fold change<− 2) were used and 8 candidate circRNAs were obtained (Fig. 1A). Among these circRNAs (Figs. S2 and S3), circEAF2 expression was the most significantly and consistently decreased in EBV-positive B lymphoma cell lines (*P* = 0.0001) and EBV + DLBCL patients (*P* = 0.0015, Fig. 1B). Particularly, when OCI-LY-10 and SU-DHL-4 cells were acutely infected with EBV, circEAF2 expression was significantly downregulated, as compared to negative control cells. During long-term EBV infection, circEAF2 expression remained low expression level, suggesting that circEAF2 downregulation was significantly associated with EBV infection in DLBCL (Fig. 1C).

CircEAF2 is produced by reverse splicing from exons 2, 3, 4, and 5 of the EAF2 gene locus (CircBase ID: hsa_circ_0066971, referred to as circEAF2, http://www.circbase.org/). To further determine the characteristics of circEAF2, we validated the putative circEAF2 junction using circRNA-specific divergent primers and Sanger sequencing (Fig. 1D). CircEAF2 was more resistant to RNase R than the liner form of EAF2 (mRNA EAF2). And the half-life of circEAF2 was longer than that of mRNA EAF2 (Fig. 1E). Moreover, the convergent primers could amplify mRNA EAF2 using both cDNA and gDNA as templates, while the divergent primers could only amplify circEAF2 using cDNA templates, but not gDNA templates (Fig. 1F). These results indicated that circEAF2 was in a circular form and functionally stable in cells.

### CircEAF2 expression is associated with EBV infection and tumor progression in DLBCL

**Fig. 2:**
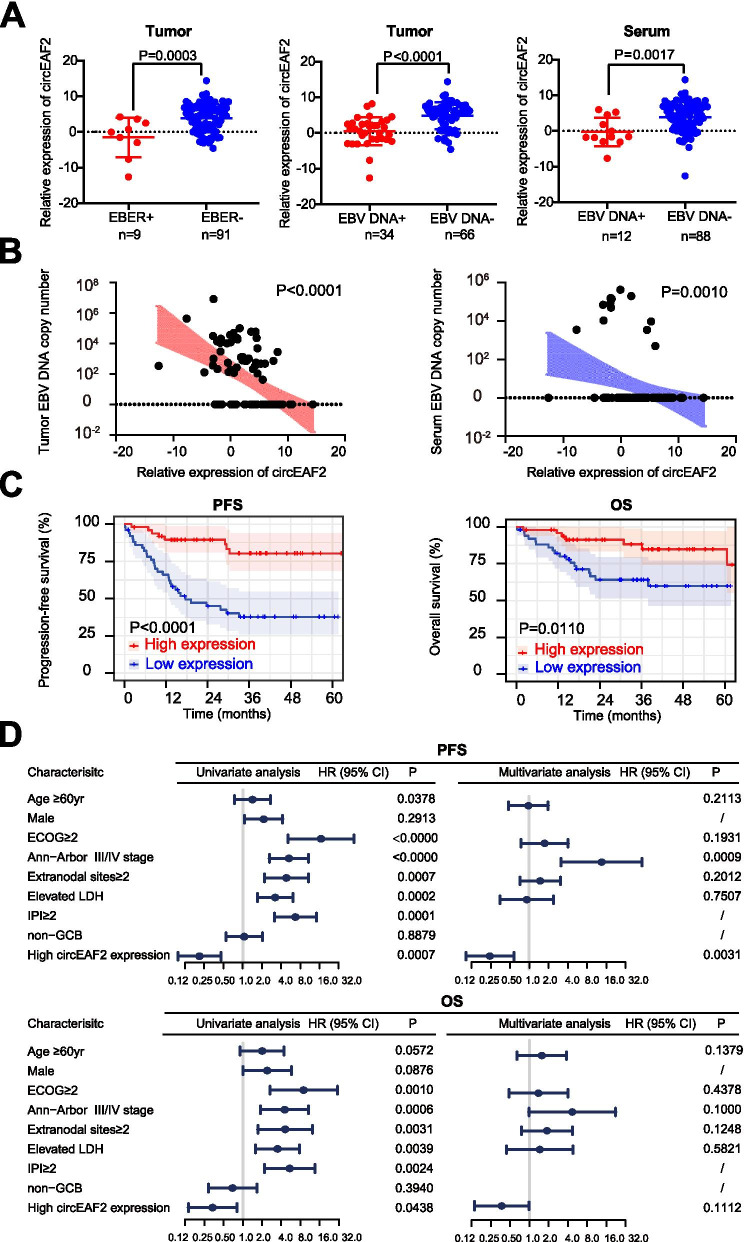
CircEAF2 is associated with EBV infection and clinical outcomes in DLBCL patients (*N* = 100). **A** Relative expression of circEAF2 according to tumor EBER-ISH, tumor EBV DNA or serum EBV DNA of DLBCL patients. Results on tumor samples was further transformed by LogE. **B** Correlations of circEAF2 expression with EBV DNA copy number in tumor and in serum. **C** Progression-free survival (PFS) and overall survival (OS) of DLBCL patients according to circEAF2 expression. **D** Forest plot showed univariate and multivariate prognostic analysis for PFS and OS of DLBCL patients. EBER, EBV-encoded RNA; ISH, in-situ hybridization; PFS, Progression-free survival; OS, overall survival; HR, Hazard ratio; CI, Confidence interval; LDH, Lactate dehydrogenase; IPI, International prognostic index; ECOG, Eastern Cooperative Oncology Group; CR, Complete remission; GCB, Germinal center B-cell

To determine whether circEAF2 was involved in EBV infection of DLBCL, the expression of circEAF2 was further assessed in tumor samples of 100 DLBCL patients. According to EBV infection status (Fig. 2A), the relative expression of circEAF2 was significantly reduced in patients with tumor EBER positivity (*P* = 0.0003), tumor EBV DNA positivity (*P* < 0.0001), and serum EBV DNA positivity (*P* = 0.0017). Moreover, circEAF2 expression showed negatively correlation with EBV DNA copy number in tumor and in serum (Fig. 2B). In addition, tumor EBV DNA was significantly associated with serum EBV DNA, poor progression-free survival (PFS) and overall survival (OS) of DLBCL patients (Fig. S4).

To evaluate the clinical relevance of circEAF2 expression in DLBCL, the median value of relative circEAF2 expression was defined as the cutoff value for dividing DLBCL patients into high- and low-expression groups (Table 1). Indeed, circEAF2 expression was negatively correlated with poor performance status (ECOG≥2, *P* = 0.0095), elevated serum LDH (*P* = 0.0152), high-risk international prognostic index (IPI 3-5, *P* = 0.0029) and low rate of complete remission (CR, *P* = 0.0206). To evaluate the prognostic value of circEAF2 expression in DLBCL, the effects of circEAF2 expression on PFS and OS of the patients were analyzed. Kaplan-Meier curves indicated that patients with low circEAF2 expression had a remarkably shorter 2-year PFS (39.2% vs 63.3%, *P* < 0.0001) and 2-year OS (52.9% vs 65.3%, *P* = 0.0110) than those with high circEAF2 expression (Fig. 2C). Moreover, univariate analysis showed that age ≥ 60 yr, ECOG≥2, advanced disease stage, multiple extranodal involvements, elevated serum LDH, and IPI score > 2 were significant predictors of inferior PFS and OS, while high circEAF2 expression was significant predictive on favorable PFS and OS (Fig. 2D). In multivariate analysis, high circEAF2 expression remained an independent favorable predictor of PFS (*P* = 0.0031, Fig. 2D). Together, circEAF2 indicated EBV infection but could counteract DLBCL progression.

**Table 1:** Clinical characteristics of DLBCL patients according to circEAF2 expression

Characteristics	Low expression	High expression	***P*** value
	*N* = 51	*N* = 49	
**Age**			0.8418
≤ 60	29 (56.9)	29 (59.2)	
> 60	22 (43.1)	20 (40.8)	
**Sex**			0.6873
Female	21 (41.2)	23 (46.9)	
Male	30 (58.8)	26 (53.1)	
**ECOG**			0.0095
0–1	22 (43.1)	34 (69.4)	
2–5	29 (56.9)	15 (30.6)	
**Ann-Arbor stage**			0.0699
I/II	18 (35.3)	27 (55.1)	
III/IV	33 (64.7)	22 (44.9)	
**Extranodal site**			0.1430
0–1	30 (58.8)	36 (73.5)	
≥ 2	21 (41.2)	13 (26.5)	
**LDH**			0.0152
Normal	16 (31.4)	28 (57.1)	
Elevated	35 (68.6)	21 (42.9)	
**IPI**			0.0029
0–2	20 (39.2)	34 (69.4)	
3–5	31 (60.8)	15 (30.6)	
**Hans**^**a**^			0.6723
Non-GCB	32 (62.7)	33 (68.8)	
GCB	19 (37.3)	15 (31.3)	
**CR**			0.0206
No	18 (35.3)	7 (14.3)	
Yes	33 (64.7)	42 (85.7)	

Values are reported as N (%) of patients unless indicated otherwise

*CR* Complete remission, *ECOG* Eastern Cooperative Oncology Group, *IPI* International Prognostic Index, *LDH* Lactic dehydrogenase, *GCB* Germinal center B-cell

^a^The calculation was based on 97 patients with available data

### CircEAF2 counteracts tumor progression of EBV + DLBCL in vitro

**Fig. 3:**
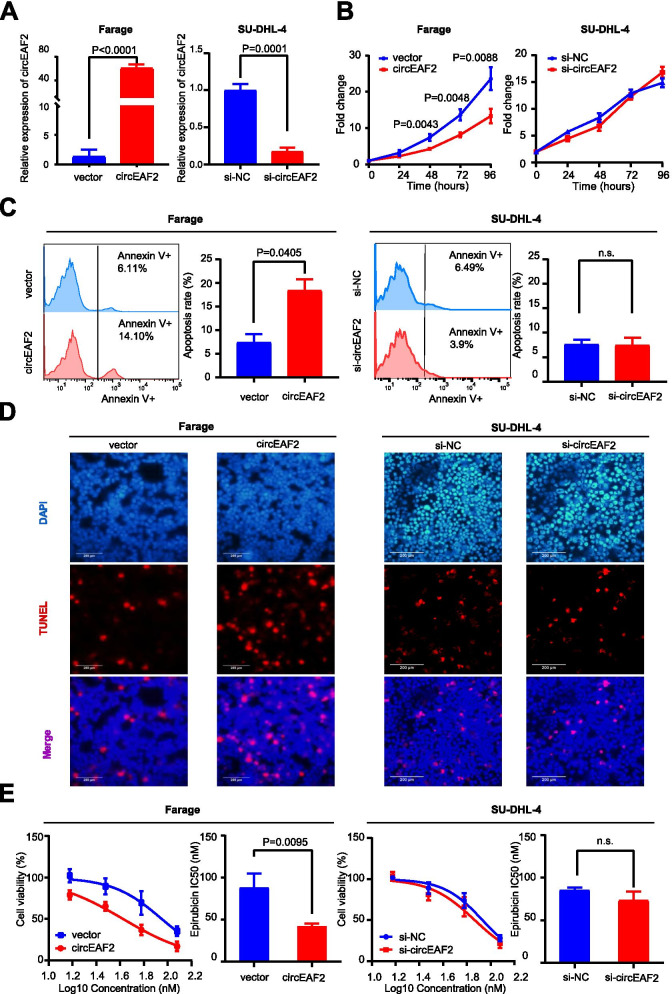
CircEAF2 counteracts the progression of EBV + DLBCL in vitro. **A** CircEAF2 overexpression in Farage cells and circEAF2 silencing in SU-DHL-4 cells. **B** CCK8 assay evaluated cell proliferation at each time point. **C** and **D** Annexin V-APC apoptosis (**C**) and TUNEL assay (**D**) detected lymphoma cell apoptosis (ratio). **E** Dose-response curves and the half maximal inhibitory concentration (IC50) for epirubicin on circEAF2-overexpressing cells or circEAF2-slienced cells. Scale bar represented 200 μm. Si-NC, Negative control siRNA; Si-circEAF2, circEAF2 siRNA; TUNEL, Terminal dexynucleotidyl transferase (TdT)-mediated dUTP nick end labeling; DAPI, 4′,6-diamidino-2-phenylindole; IC50, Half maximal inhibitory concentration. Data were shown as the mean ± S.D. of three experiments

To examine the biological functions of circEAF2 on EBV + DLBCL, EBV-positive B lymphoma cells (Farage cells) were stably transfected with lentiviruses using circEAF2 plasmid and showed significantly higher expression of circEAF2 than those transfected with vector (*P* < 0.0001). EBV-negative B lymphoma cells (SU-DHL-4 cells) were transfected with circEAF2 siRNA and showed significantly lower expression of circEAF2 than those transfected with control siRNA (*P* < 0.0001, Fig. 3A). After 48-h, ectopic circEAF2 expression resulted in a significant decrease in Farage cell proliferation, as compared to vector-transfected cells (Fig. 3B). Flow cytometry (Fig. 3C) and TUNEL assay (Fig. 3D) revealed that circEAF2 overexpression induced more apoptosis in Farage cells. Moreover, increased expression of circEAF2 sensitized Farage cells to epirubicin, with a significant decrease in the IC50 value, as compared to vector-transfected cells (42.4 ± 3.0 nM vs 88.2 ± 16.7 nM, *P* = 0.0095; Fig. 3E). In contrary, molecular silencing of circEAF2 had no significant effect on SU-DHL-4 cell proliferation, apoptosis and sensitivity to epirubicin (Fig. 3B-E). These data showed that circEAF2 could exert a protective role in EBV + DLBCL cells through promoting cell apoptosis and enhancing chemosensitivity.

### CircEAF2 functions as a sponge of miR-BART-19 in EBV + DLBCL

**Fig. 4:**
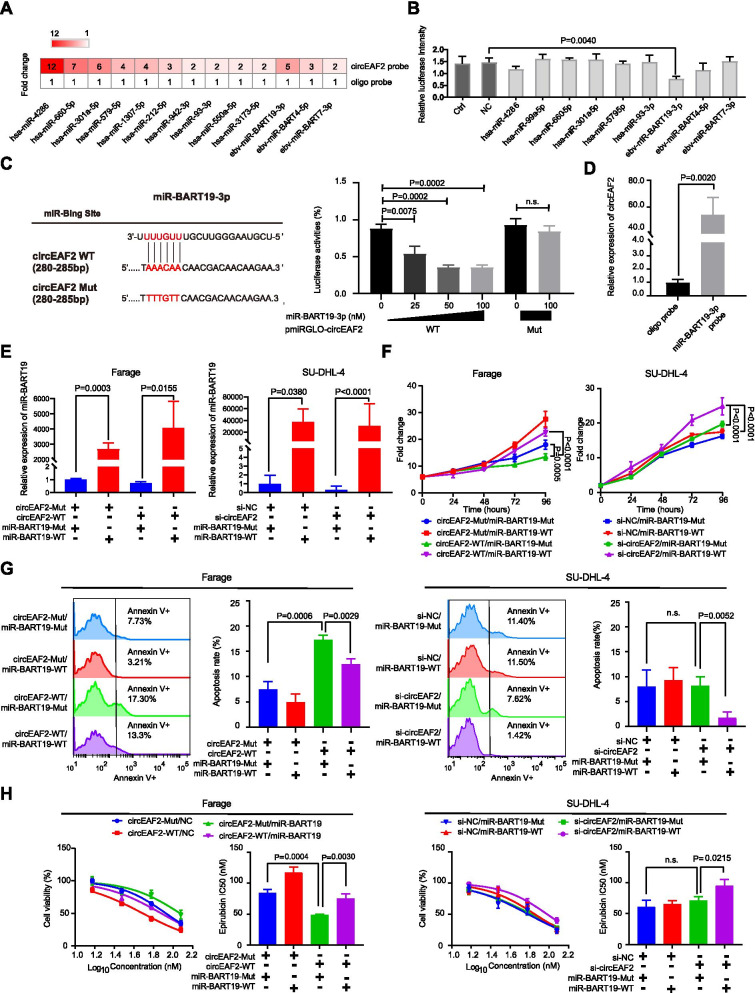
CircEAF2 functions as a sponge of miR-BART19-3p in EBV + DLBCL. **A** CircEAF2-interacting miRNAs profiles were pulled by the biotinylated-circEAF2 probe and compared with oligo probe. **B** The binding ability of miRNAs was further confirmed by circEAF2 luciferase reporter. The groups transfected wild-type circEAF2 luciferase reporter without miRNA mimics and scramble mimics were used as control group (Ctrl) and negative control group (NC), respectively. **C** Left panel: Schematic model showed the putative-binding sites for miR-BART19-3p and circEAF2. Right panel: Luciferase assay was performed in HEK293T cells transfected with the wild-type or mutant circEAF2 luciferase reporter and the indicated concentrations of miR-BART19-3p mimics. Results were normalized to a Renilla transfection control. **D** Enrichment of circEAF2 was pulled down by biotinylated-miR-BART19-3p. **E** Overexpression of miR-BART19-3p in Farage and SU-DHL-4 cells. Cell proliferation (**F**), apoptosis assay (**G**), and epirubicin sensitivity (**H**) were performed after co-transfection of miR-BART19-3p mimics with circEAF2 plasmid in Farage cells or circEAF2 siRNA in SU-DHL-4 cells. miR-BART19-WT, miR-BART19-3p mimics; miR-BART19-Mut, miR-BART19-3p mutants; Data were shown as the mean ± S.D. of three experiments

As circRNAs mostly function as miRNA sponges to regulate downstream genes, we next performed biotinylated-circEAF2 probe pull-down assay and miRNA screening to identify circEAF2-interacting miRNAs. As shown in Fig. 4A, the specific enrichment of human miRNAs (miR-4286, miR-660, miR-301a, miR-579, miR-1307, miR-212, miR-942, miR-93, miR-550, miR-3173) and EBV-encoded miRNAs (miR-BART19-3p, miR-BART4, miR-BART7) were detected in the biotinylated-circEAF2 probe, as compared to the oligo probe in Farage cells. Among these miRNAs, only EBV-encoded miR-BART19-3p mimics could significantly reduce the circEAF2 luciferase reporter activity of wild-type circEAF2 in a dose-dependent manner, but not mutant circEAF2 (Fig. 4B and C). The sponge effect of circEAF2 on miR-BART19-3p was further confirmed by pulling down biotinylated-miR-BART19-3p (Fig. 4D). CircEAF2 was abundantly pulled down by biotinylated-miR-BART19-3p probe rather than oligo probe in Farage cells.

To further investigate the functional interaction between circEAF2 and miR-BART19-3p, rescue experiments were conducted by co-transfecting miR-BART19-3p mimics (miR-BART19-WT) with circEAF2 plasmid (circEAF2-WT) in Farage cells (*P* = 0.0155) or circEAF2 siRNA (si-circEAF2) in SU-DHL-4 cells (*P* < 0.0001; Fig. 4E). As expected, miR-BART19-3p mimics (miR-BART19-WT) could partially attenuate inhibition of Farage cell proliferation (Fig. 4F), induction of apoptosis (Fig. 4G) and chemosensitivity to epirubicin (Fig. 4H) mediated by circEAF2 overexpression. In contrary, only after circEAF2 silencing in SU-DHL-4 cells, miR-BART19-WT significantly promoted SU-DHL-4 cell proliferation (Fig. 4F), inhibited apoptosis (Fig. 4G) and chemosensitivity to epirubicin (Fig. 4H). These data demonstrated that circEAF2 could function as a sponge for EBV-encoded miR-BART19-3p to modulate EBV + DLBCL progression.

### CirEAF2 regulates the Wnt signaling pathway through miR-BART19-3p/ APC/β-catenin axis

**Fig. 5:**
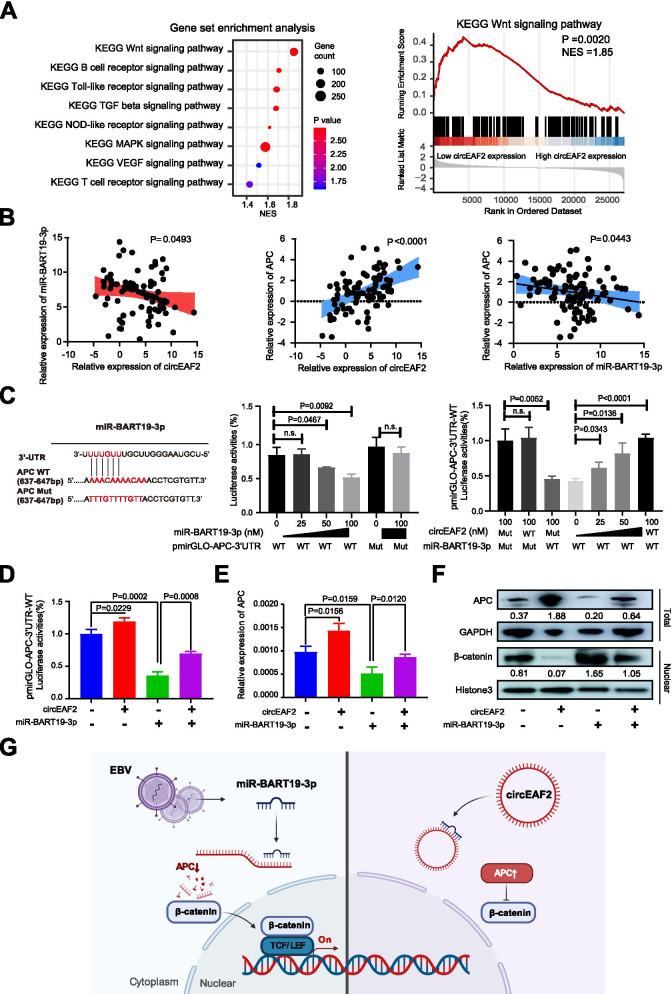
CircEAF2 functions as a sponge of miR-BART19-3p in EBV + DLBCL. **A** Gene set enrichment analysis (GSEA) according to circEAF2 expression. **B** Correlation of miR-BART19-3p expression with circEAF2 expression, APC expression with circEAF2 expression, APC expression with miR-BART19-3p expression in DLBCL tumor samples. Results on tumor samples was further transformed by LogE. **C** Left panel: Schematic model showed the putative-binding sites for miR-BART19-3p and circEAF2. Middle panel: Luciferase assay was performed in HEK293T cells transfected with the wild-type or mutant APC luciferase reporter and the indicated concentrations of miR-BART19-3p mimics. Right panel: APC-3’UTR luciferase reporter assay was evaluated in HEK293T cells co-transfected with mutant circEAF2 and miR-BART19-3p mimics, or with indicated concentrations of wild-type circEAF2 and miR-BART19-3p mimics. **D** APC-3’UTR luciferase reporter assay was evaluated in Farage cells co-transfected with wild-type circEAF2 and with miR-BART19-3p mimics. Results were normalized to a Renilla transfection control. **E** Relative mRNA expression of APC in Farage cells transfected with wild-type circEAF2 and miR-BART19-3p mimics. **F** Immunoblot assay of APC and nuclear β-catenin proteins in Farage cells transfected with wild-type circEAF2 and miR-BART19-3p mimics. Numbers showed quantification of relative protein amount. GAPDH was used as the control of total protein. Histone3 was used as the endogenous control for nuclear protein. **G** Schematic model showed the regulator network of circEAF2/miR-BART19-3p/APC/β-catenin in EBV + DLBCL. Created with BioRender.com. GSEA, Gene set enrichment analysis; KEGG, Kyoto Encyclopedia of Genes and Genomes. Data were shown as the mean ± S.D. of three experiments

To investigate the underlying mechanism of circEAF2 on EBV + DLBCL, we analyzed RNA sequencing data to identify DEGs between low circEAF2 expression group and high circEAF2 expression group. GSEA revealed Wnt signaling pathway as the most significantly altered pathway (*P* = 0.0020, NES = 1.85, Fig. 5A). Previous reports have shown that miR-BART19-3p promotes EBV-negative cell proliferation and regulates the expression of APC, a key regulator of the Wnt signaling pathway [21–23]. We assessed the expression of circEAF2, miR-BART19-3p and APC in tumor samples of DLBCL by qRT-PCR (Fig. 5B) and found significant negative correlation between circEAF2 and miR-BART19-3p (*P* = 0.0493), positive correlation between circEAF2 and APC (*P* < 0.0001), and negative correlation between miR-BART19-3p and APC (*P* = 0.0443). Therefore, we proposed that APC could be a target of circEAF2/miR-BART19-3p regulatory network in EBV + DLBCL. The putative binding sites of APC with miR-BART19-3p were further tested in a dual-luciferase assay. The results showed that APC-3’UTR luciferase reporter activity was significantly inhibited by miR-BART19-3p mimics in a dose-dependent manner, but not by circEAF2. However, circEAF2 rescued the inhibition of miR-BATR19 on APC-3’UTR luciferase reporter activity in a dose-dependent manner (Fig. 5C). In Farage cells, APC-3’UTR luciferase reporter activity was significantly decreased by miR-BART19 mimics and partially rescued by co-transfection with circEAF2 (Fig. 5D). These findings confirmed that circEAF2 sponges miR-BART-19 to regulate APC expression.

As confirmed in qRT-PCR (Fig. 5E) and western blotting (Fig. 5F), the expression of APC was upregulated in circEAF2-overexpressed Farage cells at both mRNA and protein levels, whereas was partially rescued by co-transfection with miR-BART19-3p. Accordingly, nuclear expression of β-catenin was decreased after circEAF2 overexpression and partially restored by miR-BART19-3p mimics (Fig. 5F). Therefore, the mechanistic analysis showed that circEAF2 modulated miR-BART19-3p/APC/β-catenin axis to counteract EBV + DLBCL progression (Fig. 5G).

### CircEAF2 counteracts tumor progression of EBV + DLBCL in vivo

**Fig. 6:**
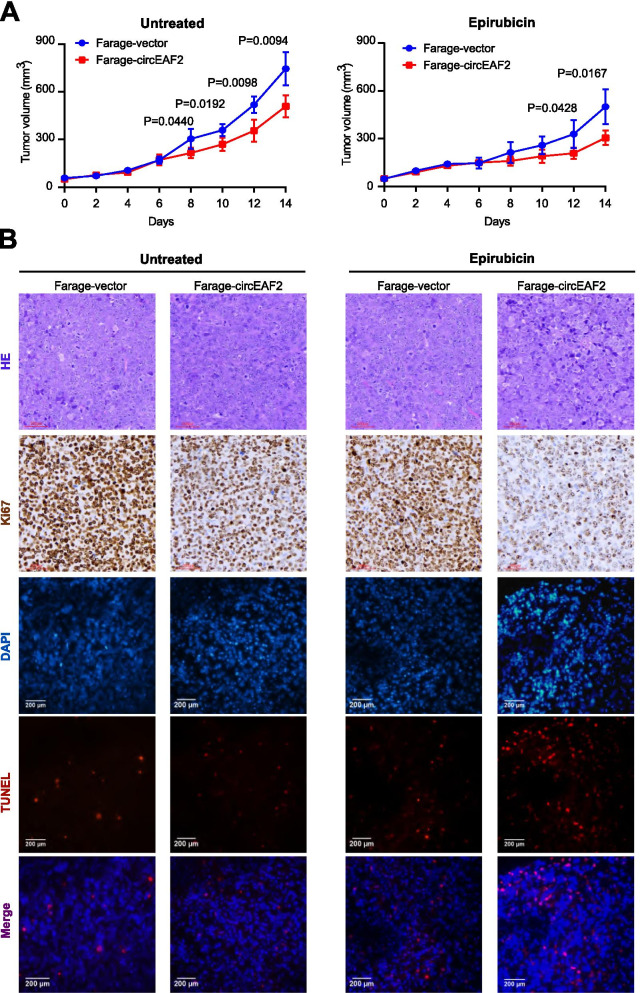
CircEAF2 attenuates the progression of EBV + DLBCL in vivo. **A** In the untreated and epirubicin group, the growth curve of xenograft tumors transfected with circEAF2 overexpression and with vector. **B** Immunohistochemistry analysis of Ki-67 and TUNEL assay of cell apoptosis in xenograft tumors transfected with circEAF2 overexpression and with vector. HE, hematoxylin-eosin staining. Untreated, the untreated group; Epirubicin, the epirubicin group; Scale bar represents 200 μm. Data were shown as the mean ± S.D. of three experiments

In order to search for in vivo evidence of circEAF2 on EBV + DLBCL progression, murine xenograft models were established with subcutaneous injection of Farage cells stably transfected with circEAF2 or vector as control. Consistent with in vitro data, reduced volume of xenograft tumors was observed in the circEAF2-overexpressing group, as compared to that of the vector group. Moreover, the circEAF2-overexpressing group treated with epirubicin showed greater reductions in tumor growth (Fig. 6A). Accordingly, immunohistochemistry study and TUNEL assay showed that the circEAF2-overexpressing tumors, treated with or without epirubicin, exhibited decreased positivity of Ki-67 and increased rate of apoptotic cells, as compared to the vector group (Fig. 6B).

### CircEAF2 may be regulated by EBV through RNA splicing

To elucidate the underlying mechanism of circEAF2 downregulation in EBV + DLBCL, RNA sequencing data were further analyzed for DEGs, according to EBV infection status and relative circEAF2 expression (Fig. S5A). In both EBV + DLBCL patients and low circEAF2 expression group, upregulated genes were identified and significantly enriched in mRNA metabolic process and RNA splicing (Fig. S5B). Considering circRNAs are generated by the backsplicing of precursor mRNA (pre-mRNA) transcripts, catRAPID was used to estimate the binding propensity of proteins with pre-mRNA EAF2. Among the upregulated genes, UPF1, RBM15B and DHX38 were predicted to have intense interaction with intronic complementary elements of pre-mRNA EAF2, suggesting the disruption of circEAF2 backsplicing formation (Fig. S5C). Moreover, relative EAF2 mRNA expression by RNA sequencing showed no difference, according to EBV infection status and relative circEAF2 expression in DLBCL (Fig. S5D). Neither upon acute nor upon long-term EBV infection, the EAF2 expression had no obvious change in SU-DHL-4 cells and OCI-LY-10 cells (Fig. S5E), suggesting that EBV infection may reduce expression of circEAF2 by RNA splicing, but not expression of linear EAF2. Together, these results supported the notion that EBV infection in DLBCL may alter RNA splicing and result in disruption of circEAF2 formation.

## Discussion

CircRNAs have important functions in oncogenesis and tumor progression [24, 25]. In DLBCL, circAPC suppresses tumor cell proliferation by sponging miR-888, upregulates APC expression, and eventually inactivates Wnt/β-catenin pathway. Clinically, high circAPC expression was associated with favorable prognosis [10]. More recent study has reported that EBV-encoded circRNAs are implicated in EBV biology and cancers [26]. However, the function and regulatory mechanism of circRNAs in EBV + DLBCL remain largely unknown.

In the present study, circEAF2 was identified as a novel circRNA significantly downregulated in EBV + DLBCL, negatively correlated with EBV infection, and indicated good clinical outcome of the patients. In vitro and in vivo studies further revealed that circEAF2 counteracted EBV + DLBCL progression through inducing lymphoma cell apoptosis and sensitizing lymphoma cell to epirubicin. Recently, several studies implicated the role of circRNAs in tumor sensitivity to chemotherapy [27]. Circ-LARP4 is downregulated and correlated with prolonged survival in osteosarcoma, due to its ability to increase tumor cell sensitivity to doxorubicin by sponging miR-424 [28]. Similar observations were reported in breast cancer, where circKDM4C suppresses tumor progression and enhances doxorubicin chemosensitivity by regulating miR-548p/PBLD axis [29]. Epirubicin is a major anthracycline cytotoxic agent for DLBCL [30]. EBV + DLBCL tends to have poor response and inferior survival upon treatment with standard anthracycline-based chemotherapy [1]. Here we demonstrated that circEAF2 overexpression was associated with favorable prognosis of the patients and good response to anthracycline-based immunochemotherapy in DLBCL. Functionally, circEAF2 overexpression sensitized EBV-positive lymphoma cells to epirubicin both in vitro and in vivo. Together, circEAF2 exhibits a tumor suppressive role and could be considered as a favorable prognostic biomarker of EBV + DLBCL.

EBV-encoded miRNAs are critically involved in EBV-associated oncogenesis and function as oncogenic drivers [4], modulating host mRNAs involved in transcription regulation, apoptosis, and cell cycle control [31]. This is, to our knowledge, the first study to explore the potential interaction between circRNA and EBV-encoded miRNA in DLBCL. Indeed, we screened oncogenic miRNAs and confirmed that circEAF2 was able to bind to EBV-encoded miR-BART19-3p. High expression of miR-BART19-3p was previously found in Hodgkin lymphoma [32] and NK/T-cell lymphoma [33]. MiR-BART19-3p is implicated in the activation of Wnt signaling pathway by targeting tumor suppressor APC, enhancing proliferation and suppressing apoptosis of EBV-infected tumor cells [21]. APC can downregulate Wnt signaling pathway by reducing the accumulation of β-catenin in the nucleus, thereby inhibit tumor growth and chemoresistance in multiple cancer types [34, 35]. Here in EBV-positive B-lymphoma cells, circEAF2 specifically targeted miR-BART19-3p, upregulated APC, and suppressed downstream β-catenin, indicative a viral miRNA-mediated mechanism underlying EBV + DLBCL progression. In EBV-associated nasopharyngeal carcinoma, circRPMS1 mediates oncogenesis through sponging multiple miRNAs [36]. Our results elucidated not only the suppressive role of circEAF2 in EBV + DLBCL progression, but also the interaction between circEAF2 and miR-BART19-3p to become a potential therapeutic target for EBV + DLBCL treatment.

Previous research indicated that circRNA formation is regulated by RNA splicing regulatory proteins, such as eIF4A3 and ADAR1 [37, 38]. In this study, we found that RNA splicing-related genes were significantly enriched in EBV + DLBCL patients with low circEAF2 expression group. Among them, UPF1, RBM15B and DHX38 were predicted to bind to intronic complementary elements of pre-mRNA EAF2. UPF1, a nonsense-mediated RNA decay factor, is critically involved in host cellular response to EBV infection [24, 25]. RBM15B, initially identified as a binding partner of the EBV mRNA export factor, mediates RNA N6-methyladenosine methylation (m6A) and regulates RNA alternative splicing [26]. DHX38, a pre-mRNA splicing-related DEAH box RNA helicase, interacts with satellite I noncoding RNA [27]. Based on these observations, one possibility is that EBV infection in DLBCL may alter RNA splicing and result in disruption of circEAF2 formation. Upon both acute and long-term EBV infection, circEAF2 expression was significantly downregulated, while linear EAF2 expression had no obvious change. These findings supported that EBV infection actively represses circEAF2 formation by RNA splicing. Another possibility is that miR-BART-19-3p indirectly represses circEAF2 through negative selection as circEAF2 low-expressing cells outcompete circEAF2 high-expressing cells. As previously reported, EBV-encoded BART miRNAs conferred a selective growth advantage to EBV-positive tumor cells in vivo [39]. Thus it is also likely that such oncogenic properties reported for BART-miRNAs could indirectly represses circEAF2 through negative selection. However, future study is needed to further illustrate the regulatory mechanisms on circEAF2 in EBV + DLBCL.

## Conclusions

Understanding the precise function of circEAF2 in the progression of EBV + DLBCL may increase our knowledge of EBV lymphomagenesis and provide clinical rationale of developing new therapeutic strategy in treating EBV-associated lymphoid malignancies.

## Supplementary Information

**Additional file 1: Table S1.** The primer sequences used for real-time quantitative PCR.

**Additional file 2: Figure S1.** Construction and verification for circEAF2 plasmid.

**Additional file 3: Table S2.** Sequence information of miRNA mimics and circ siRNA.

**Additional file 4: Table S3.** Sequence information of biotinylated probes.

**Additional file 5: Figure S2.** Verification of EBV-related circRNAs in B-lymphoma cell lines.

**Additional file 6: Figure S3.** Verification of EBV-related circRNAs in tissues samples of 54 DLBCL patients.

**Additional file 7: Figure S4.** Tumor EBV DNA is associated with serum EBV DNA an d clinical outcomes in DLBCL patients.

**Additional file 8: Figure S5.** CircEAF2 may be regulated by EBV infection through RNA splicing.

## Data Availability

Further information and requests for reagents and resources should be directed to and will be made available by the lead contact Wei-li Zhao (zhao.weili@yahoo.com) upon reasonable request.

## Abbreviations

EBV: Epstein-Barr virus
DLBCL: Diffuse large B-cell lymphoma
miRNA: microRNA
CircRNA: Circular RNA
BHRF1: BamHI fragment H rightward open reading frame 1
BART: Bam HI-A region rightward transcript
WHO: World Health Organization
ATCC: American type culture collection
R-CHOP: Rituximab combined with cyclophosphamide, doxorubicin, vincristine and prednisone
EBER: EBV-encoded RNA
ISH: In-situ hybridization
gDNA: Genomic DNA
cDNA: Complement deoxyrubonucleic acid
LDH: Lactate dehydrogenase
IPI: International prognostic index
ECOG: Eastern Cooperative Oncology Group
CR: Complete remission
GCB: Germinal center B-cell
OS: Overall survival
PFS: Progression-free survival
HR: Hazard ratio
CI: Confidence interval
PCR: Polymerase chain reaction
qRT-PCR: Quantitative real-time PCR
CCK-8: Cell counting Kit-8
TUNEL: Terminal dexynucleotidyl transferase (TdT)-mediated dUTP nick end labeling
DAPI: 4′,6-diamidino-2-phenylindole
IC50: Half maximal inhibitory concentration
DEGs: Differentially expressed genes
GSEA: Gene set enrichment analysis
KEGG: Kyoto Encyclopedia of Genes and Genomes
