# Supplementary material for: CircEAF2 counteracts Epstein-Barr virus-positive diffuse large B-cell lymphoma progression via miR-BART19-3p/APC/β-catenin axis
Source: Mol Cancer. 2021 Dec 1;20:153. doi: 10.1186/s12943-021-01458-9 (PMC8638185; doi:10.1186/s12943-021-01458-9)
Supplement: Supplementary file 2 — Additional file 2: Figure S1. Construction and verification for circEAF2 plasmid. [file 12943_2021_1458_MOESM2_ESM.pdf]

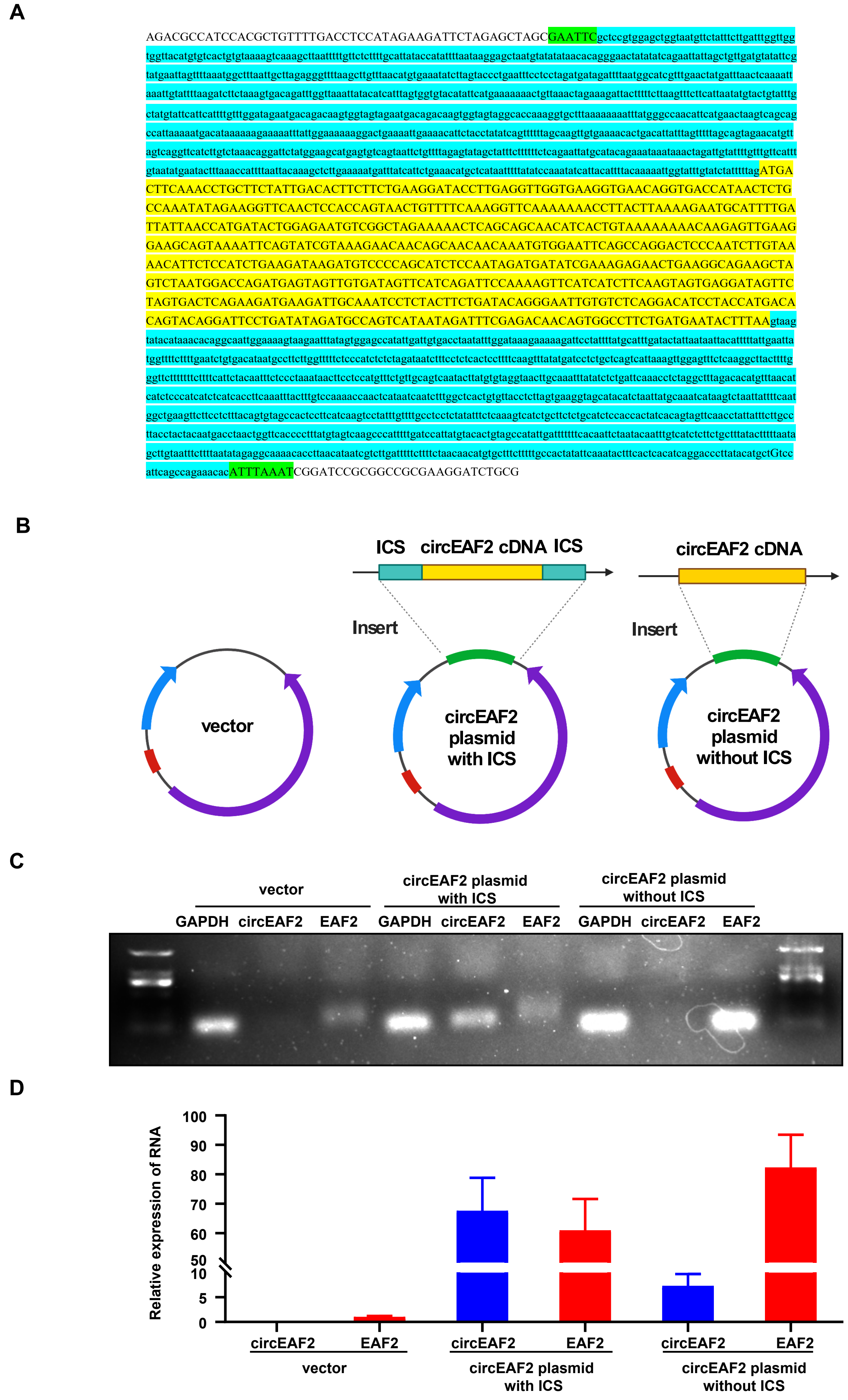

Figure S1. Construction and verification for circEAF2 plasmid.

(A) The designed sequences for circEAF2 plasmid. The yellow region indicates the mature sequence of circEAF2 cDNA. The blue region indicates the intronic complementary sequence (ICS) of *EAF2* (ENSG00000145088) and the green region indicates the restriction enzyme cutting site.**(B)** Schematic model shows the lentiviral plasmid constructs of vector, circEAF2 plasmid with ICS and circEAF2 plasmid without ICS. qPCR assay divergent or convergent primers indicated the existence of circEAF2 or linear EAF2, respectively. As shown in nucleic acid electrophoresis **(C)** and relative expression of RNA **(D)**, compared with vector, circEAF2 plasmid with ICS notably increased the expression of circular EAF2, while circEAF2 plasmid without ICS only increased the expression of linear RNA, but not circular RNA. ICS, intronic complementary sequence.
