# Supplementary material for: CircEAF2 counteracts Epstein-Barr virus-positive diffuse large B-cell lymphoma progression via miR-BART19-3p/APC/β-catenin axis
Source: Mol Cancer. 2021 Dec 1;20:153. doi: 10.1186/s12943-021-01458-9 (PMC8638185; doi:10.1186/s12943-021-01458-9)
Supplement: Supplementary file 5 — Additional file 5: Figure S2. Verification of EBV-related circRNAs in B-lymphoma cell lines. [file 12943_2021_1458_MOESM5_ESM.pdf]

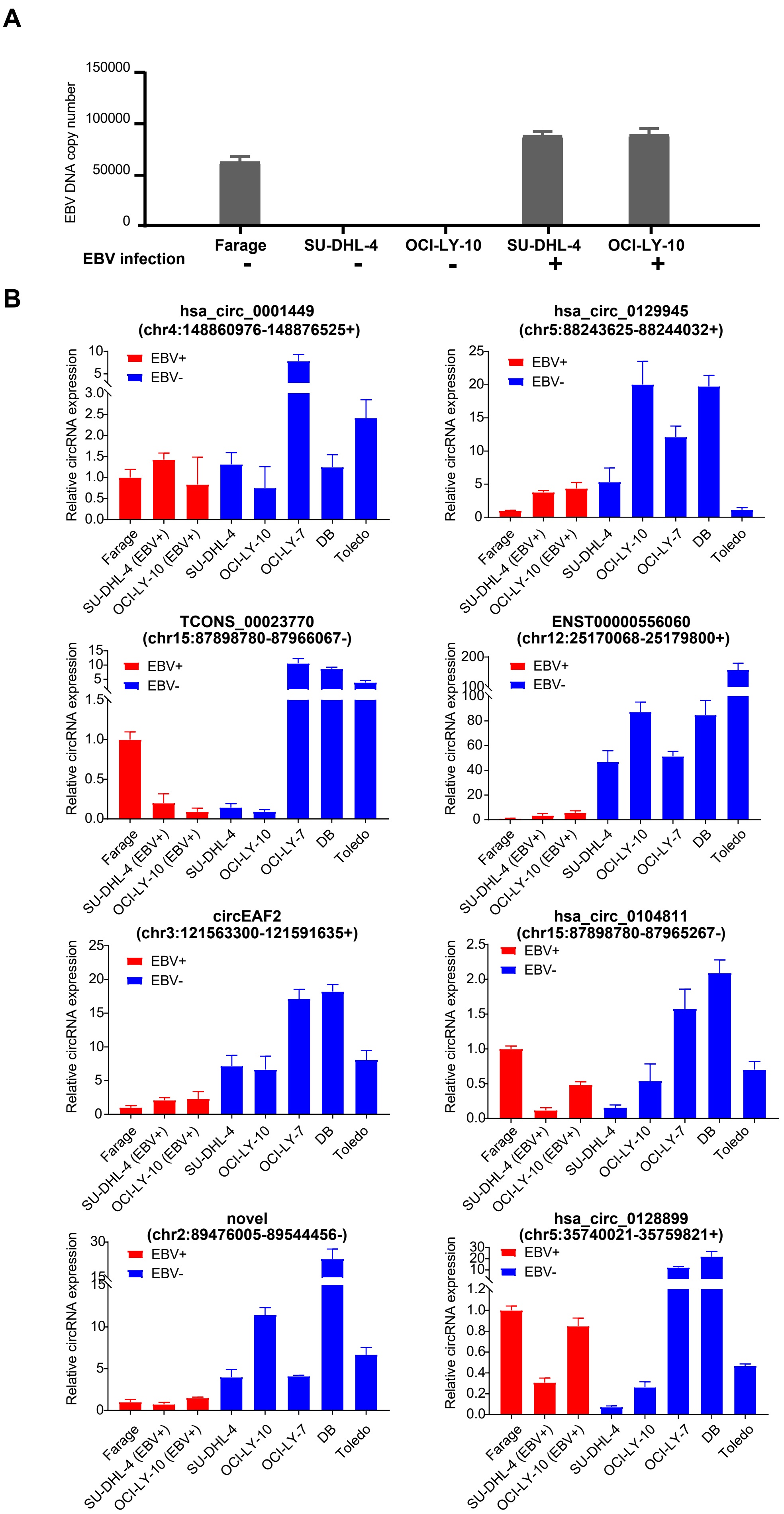

Figure S2. Verification of EBV-related circRNAs in B-lymphoma cell lines.

(A). B-lymphoma cell line (SU-DHL-4 and OCI-LY-10) were successfully infected with EBV and showed significantly higher of EBV DNA copy number. Representative sample of EBV-infected cell line (Farage) was used as positive control. (B). qRT-PCR was performed to verify the relative expression of the eight candidate circRNAs in B-lymphoma cell lines with or without EBV infection. All the data are shown as the mean ± S.D.
