# Supplementary material for: CircEAF2 counteracts Epstein-Barr virus-positive diffuse large B-cell lymphoma progression via miR-BART19-3p/APC/β-catenin axis
Source: Mol Cancer. 2021 Dec 1;20:153. doi: 10.1186/s12943-021-01458-9 (PMC8638185; doi:10.1186/s12943-021-01458-9)
Supplement: Supplementary file 6 — Additional file 6: Figure S3. Verification of EBV-related circRNAs in tissues samples of 54 DLBCL patients. [file 12943_2021_1458_MOESM6_ESM.pdf]

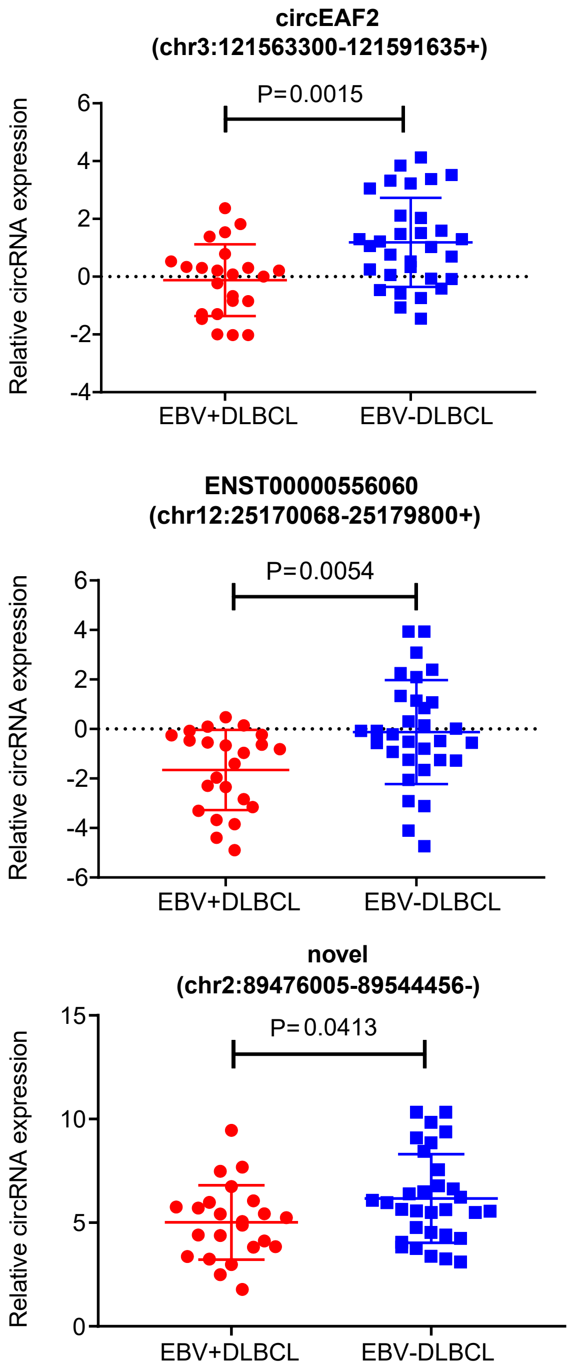

Figure S3. Verification of EBV-related circRNAs in tissues samples of 54 DLBCL patients.

Among the three circRNAs deregulated in EBV-positive B cell lines, the Mann-Whitney test was used to detect the relative expression level of the indicated circRNAs in 23 EBV+DLBCL tumor tissues compared to 31 EBV-DLBCL tumor tissues.
