# Supplementary material for: CircEAF2 counteracts Epstein-Barr virus-positive diffuse large B-cell lymphoma progression via miR-BART19-3p/APC/β-catenin axis
Source: Mol Cancer. 2021 Dec 1;20:153. doi: 10.1186/s12943-021-01458-9 (PMC8638185; doi:10.1186/s12943-021-01458-9)
Supplement: Supplementary file 7 — Additional file 7: Figure S4. Tumor EBV DNA is associated with serum EBV DNA an d clinical outcomes in DLBCL patients. [file 12943_2021_1458_MOESM7_ESM.pdf]

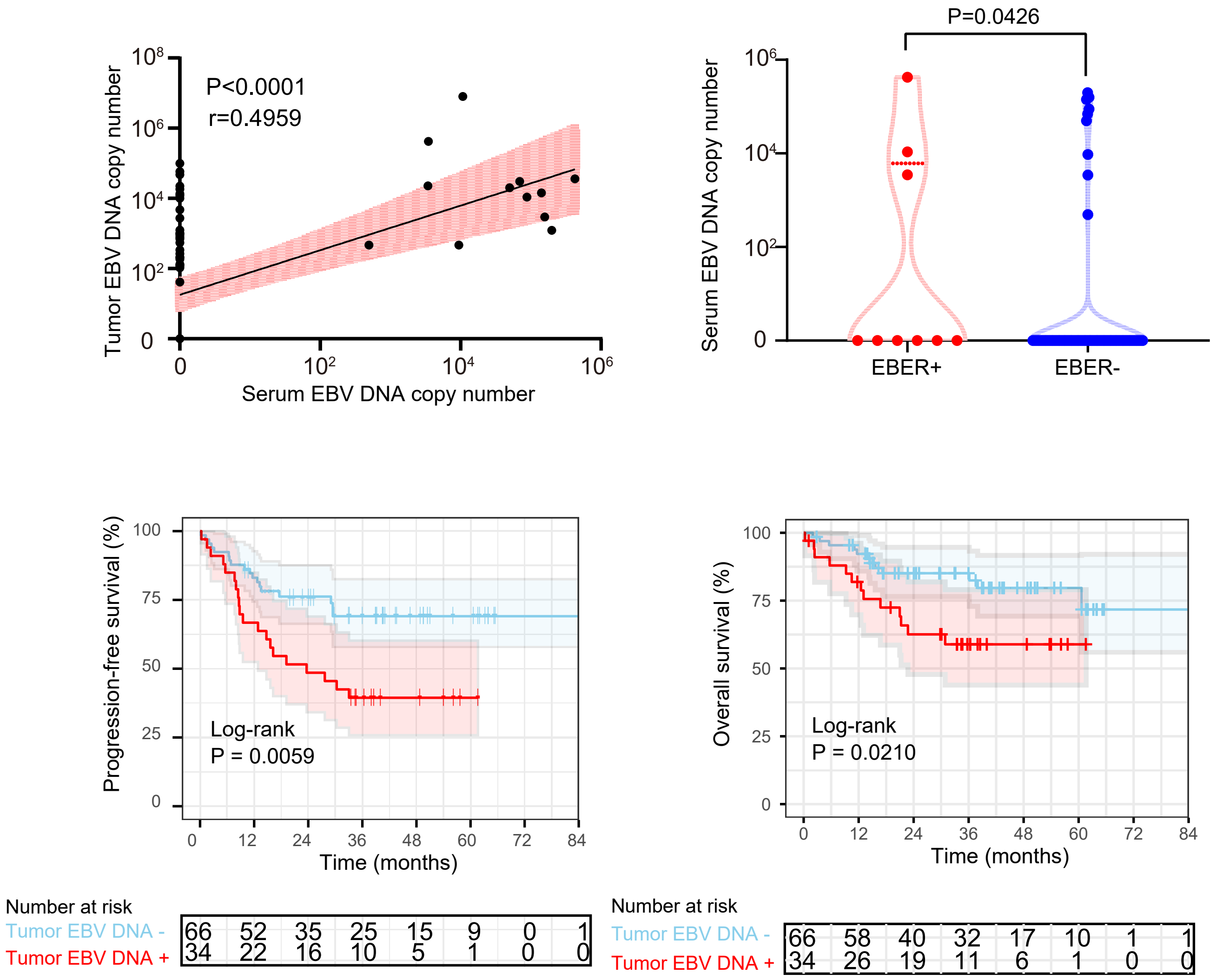

Figure S4. Tumor EBV DNA is associated with serum EBV DNA and clinical outcomes in DLBCL patients.

(**A**) Correlations of serum EBV DNA load with tumor EBV DNA load or tumor EBER-ISH in DLBCL patients. (**B**) Progression-free survival (PFS) and overall survival (OS) of DLBCL patients according to tumor EBV DNA.
