# Supplementary material for: CircEAF2 counteracts Epstein-Barr virus-positive diffuse large B-cell lymphoma progression via miR-BART19-3p/APC/β-catenin axis
Source: Mol Cancer. 2021 Dec 1;20:153. doi: 10.1186/s12943-021-01458-9 (PMC8638185; doi:10.1186/s12943-021-01458-9)
Supplement: Supplementary file 8 — Additional file 8: Figure S5. CircEAF2 may be regulated by EBV infection through RNA splicing. [file 12943_2021_1458_MOESM8_ESM.pdf]

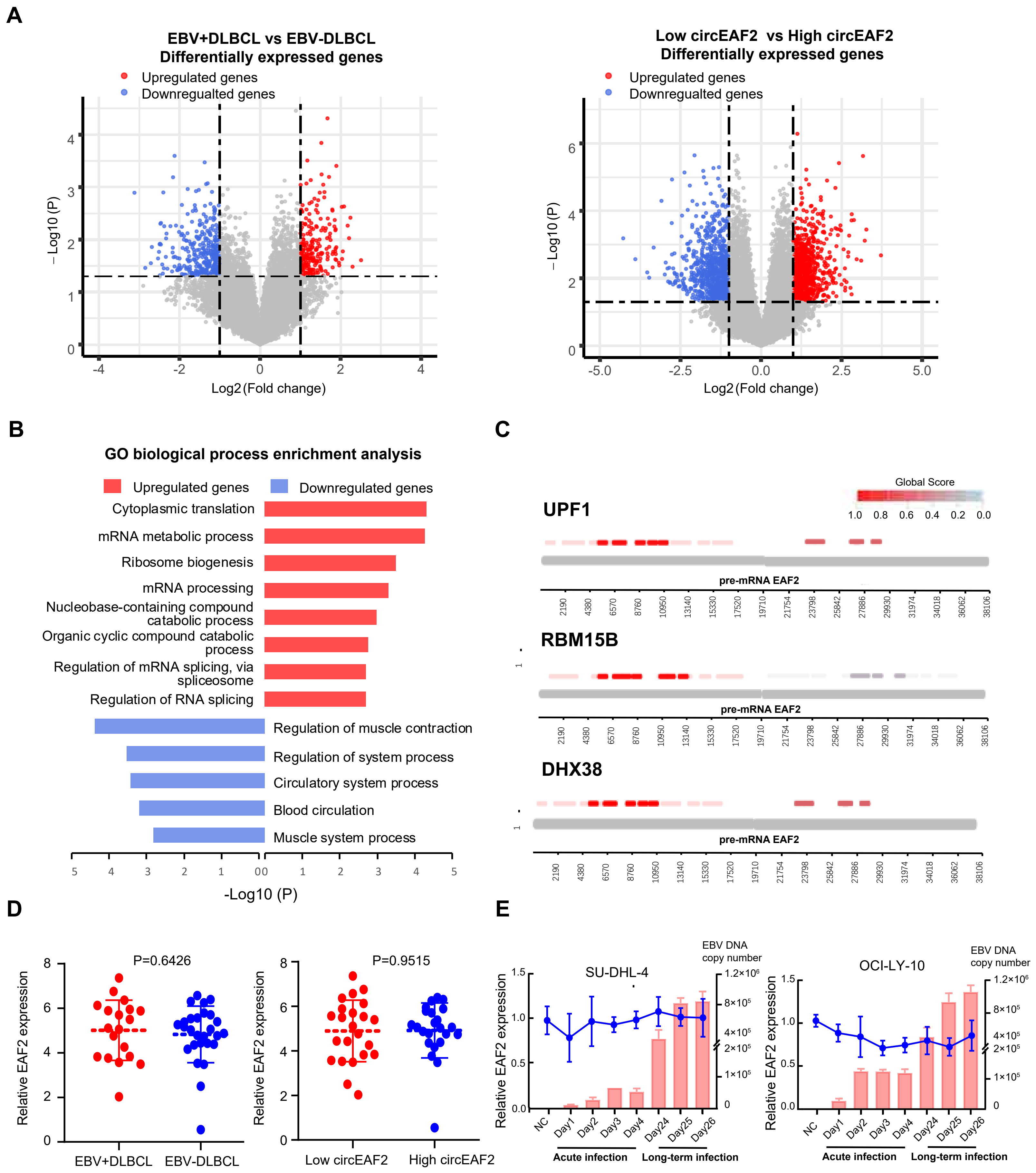

Figure S5. CircEAF2 may be regulated by EBV infection through RNA splicing.

(A) Volcano plot showed the differential expressed genes according to EBV infection status and relative circEAF2 expression, analyzed by RNA sequencing. **(B)** GO biological process enrichment analysis of upregulated genes (red) or downregulated genes (blue) in EBV+DLBCL patients and low circEAF2 expression group. **(C)** The signal localization plot represented the localization of the signal along the pre-mRNA sequence (EAF2) colored according to the global score value, using catRAPID method. **(D)** Relative expression of EAF2, according to EBV infection status and relative circEAF2 expression of DLBCL patients, obtained by RNA sequencing. **(E)** Relative expression of EAF2 (line graph) and EBV DNA copy number (bar graph) in SU-DHL-4 and OCI-LY-10 cells during acute EBV infection or long-term EBV infection, as compared to those without EBV infection (negative control, NC).
